# Estimated costs for Duchenne muscular dystrophy care in Brazil

**DOI:** 10.1186/s13023-023-02767-6

**Published:** 2023-06-22

**Authors:** Nayê Balzan Schneider, Erica Caetano Roos, Ana Lúcia Portella Staub, Isabela Possebon Bevilacqua, Ana Carolina de Almeida, Tamiê de Camargo Martins, Natalia Bergamelli Ramos, Priscilla Loze, Jonas Alex Morales Saute, Ana Paula Beck da Silva Etges, Carisi Anne Polanczyk

**Affiliations:** 1 National Institute of Science and Technology for Health Technology Assessment (IATS)- CNPq/Brazil (project: 465518/2014-1), Porto Alegre, Brazil; 2 Programa de Pós-Graduação em Epidemiologia, Universidade Federal do Rio Grande do Sul, Porto Alegre, Brazil; 3 Programa de Pós-Graduação em Engenharia de Produção, Universidade Federal do Rio Grande do Sul, Porto Alegre, Brazil; 4 Centro de Pesquisa Clínica, Hospital de Clínicas de Porto Alegre, Neurogenética, Porto Alegre, Brazil; 5 Produtos Roche Químicos e Farmacêuticos S/A, São Paulo, SP Brazil; 6 Hospital de Clínicas de Porto Alegre, Porto Alegre, Brazil; 7 Programa de Pós-Graduação em Medicina: Ciências Médicas, Faculdade de Medicina, Universidade Federal do Rio Grande do Sul, Porto Alegre, Brazil; 8 Departamento de Medicina Interna, Faculdade de Medicina, Universidade Federal do Rio Grande do Sul, Porto Alegre, Brazil; 9 Serviço de Genética Médica, Hospital de Clínicas de Porto Alegre, Porto Alegre, Brazil; 10 Serviço de Neurologia, Hospital de Clínicas de Porto Alegre, Porto Alegre, Brazil; 11 School of Technology, Pontifícia Universidade Católica do Rio Grande do Sul, Porto Alegre, Brazil; 12 Hospital Moinhos de Vento, Porto Alegre, Rio Grande do Sul Brazil

**Keywords:** Cost of illness, Muscular dystrophy, Duchenne, Neuromuscular diseases, Rare diseases

## Abstract

**Background:**

The economic burden of rare diseases on health systems is still not widely measured, with the generation of accurate information about the costs with medical care for subjects with rare diseases being crucial when defining health policies. Duchenne Muscular Dystrophy (DMD) is the most common form of muscular dystrophy, with new technologies recently being studied for its management. Information about the costs related to the disease in Latin America is scarce, and the objective of this study is to evaluate the annual hospital, home care and transportation costs per patient with DMD treatment in Brazil.

**Results:**

Data from 27 patients were included, the median annual cost per patient was R$ 17,121 (IQR R$ 6,786; 25,621). Home care expenditures accounted for 92% of the total costs, followed by hospital costs (6%) and transportation costs (2%). Medications and loss of family, and patient’s productivity are among the most representative consumption items. When disease worsening due to loss of the ability to walk was incorporated to the analysis, it was shown that wheelchair users account for an incremental cost of 23% compared with non-wheelchair users.

**Conclusions:**

This is an original study in Latin America to measure DMD costs using the micro-costing technique. Generating accurate information about costs is crucial to provide health managers with information that could help establish more sustainable policies when deciding upon rare diseases in emerging countries.

**Supplementary Information:**

The online version contains supplementary material available at 10.1186/s13023-023-02767-6.

## Background

Duchenne Muscular Hystrophy (DMD) is a X-linked inherited genetic disease caused by mutations in the *DMD* gene. Variants leading to loss of dystrophin function, a product of this gene, modify the structure of the muscle cell that is progressively replaced by fibrosis and fatty tissue, resulting in a clinical setting of progressive muscle weakness. Most boys with DMD inherit the condition from their mother; however, any *de novo* mutations are reported in approximately 1/3 of the cases. Although there are variations in the clinical evolution, the condition leads to early loss of the ability to walk, followed by cardiac and respiratory complications throughout life [1–3]. In a recent meta-analysis, the global overall mean prevalence of the disease was 19.8 cases per 100,000 male live births (95% CI: 16.6–23.6) [4]. Data from a single-center study in the state of Ceará indicate a prevalence of 0.44 cases per 100,000 (95% CI: 0.31–0.6) [5]; however, the overall prevalence of DMD in Brazil is unknown. A recent study from Brazil suggested that the molecular epidemiology of DMD cases in Brazil is similar to that from other regions worldwide, with major deletions and duplications identified in approximately 70% of cases, followed by nonsense variants in 13% of cases [6].

Difficulty to walk and need for wheelchair during adolescence, or before that, make patients with DMD highly dependent on their relatives. Life expectancy decreases with age, with a median life expectancy of 28 years for patients born after 1990 [7]. Advances in the care related to cardiac and respiratory problems have increased survival and, consequently, costs associated with disease care [3, 8, 9]. Treatment for DMD usually includes the continuous use of corticosteroids associated with other outpatient follow-up interventions from multiple professionals at reference sites, which delays the disease progression and allows an improved quality of life [10, 11].

Ataluren is a new drug recommended for patients with nonsense mutations in *DMD* gene, which is present in 10-15% of patients. It is not currently available in the Brazilian Unified Health System [*Sistema Único de Saúde* (SUS)], and it has been approved by the Brazilian National Health Surveillance Agency [*Agência Nacional de Vigilância Sanitária* (Anvisa)] in 2019 for male children who are able to walk, aged ≥ 5 years old, with DMD due to this genetic variant. Other medications have been recently approved in the United States for patients with specific mutations: eteplirsen (in 2016), golodirsen and viltolarsen (in 2020), and casimersen (in 2021). However, these medications are not approved by Anvisa for use in Brazil [12].

The lack of accurate information about the costs related to rare diseases, specifically for DMD, limits the ability to better manage the resources available for treating such diseases [13]. Generating costs and detailed information is crucial to target efficient use of resources to manage patients with this rare condition in the health systems [14].

The objective of this study is to evaluate the annual hospital, home care and transportation costs per patient for the treatment of DMD in Brazil.

## Methods

The study is characterized as a cohort analysis, with retrospective collection of the patients’ data, and the cost analyses using microcosting techniques are under the perspective of the public health system and of the patients (family).

Patients diagnosed with DMD treated throughout 2019 at Hospital de Clínicas de Porto Alegre (HCPA), who were able (or had a relative who was able) to understand and answer the study questionnaire and with no other chronic diseases impacting morbidity and mortality (with a life expectancy < 2 years), were deemed eligible. Only patients who agreed with the General Personal Data Protection Law [*Lei Geral de Proteção de Dados* (LGPD)] were invited to participate in the study. After accepting and signing the Informed Consent Form (ICF), patients were enrolled in the study. This study was submitted to and approved by the Ethical Committee of HCPA (CAAE: 54235721.9.0000.5327).

### Hospital and transportation costs

To survey all hospital and transportation costs, the steps from the Time-driven Activity-based Costing (TDABC) method were followed [14, 15], including: mapping of the flow of care with the main activities to which the patient is submitted to; identification of all resources and departments used by the patient; estimation of the overall expenses of every resource identified in the flow of care; estimation of the hourly capacity of every resource or department, and unit cost rate (UCR) calculation; analysis of the time spent on the patient for every resource, and structuring of the time and costs equations; cost per patient calculation; and statistical analyses.

### Mapping of the flow of care for the main activities to which the patient is submitted

The mapping of the flow of care was performed by a multidisciplinary team, including physical therapists and physicians from the neurology and medical genetics services of the site participating in the project, as well as researchers from the health technologies evaluation area.

### Identification of all hospital resources and departments used by the patient

Based on the review of clinical records in the electronic medical files from patients enrolled in the study, the locations at the reference site where the patient is treated were identified, as well as the equipment and workers involved in the activities to which patients are submitted.

### Estimation of the total cost of every resource identified in the hospital flow of care

For each resource identified, annual mean costs were calculated. For the physical structure of the hospital, site data of costs from the departments where the patient received care were surveyed with hospital financial department, considering fixed costs of depreciation, energy, supporting materials, taxes, and system licenses. For labor-costs, the data from 2019 was used, including charges by mean professional class from the institution.

### Estimation of the hourly capacity of every resource or department and unit cost rate calculation

The capacity of the ambulatory units where patients with DMD were treated were estimated considering the availability of rooms and service teams and the utilization of the installed capacity. For labor, the workload contracted throughout one month was used. With the information on cost per resource and capacity it is possible to calculate the cost-capacity-rate (CCR) for each resource.

### Analysis of the time spent on the patient for every resource and structuring of the time and cost equations

The mean time every worker spends involved with the consultations and procedures performed on patients with DMD was estimated based on reports from healthcare professionals.

### Hospital and transportation cost calculation

In order to evaluate the individual cost per patient, the time spent for each resource was multiplied by the time CCR and, subsequently, by the costs with medications and exams. For medications, the individual consumption was extracted from the medical chart, and the cost considered was the one recorded in 2019 in the Health Prices Bank [*Banco de Preços em Saúde* (BPS)] [16]. Exams were computed from the identification in the clinical records of the exams, with the costs recorded at the site being used as the basis for the financial information.

Finally, the cost with transportation to the reference site per patient was estimated. For residents in the city of Porto Alegre, two round-trip public transportation tickets were accounted for each consultation when transportation costs were not informed by the patient. For residents of other cities, the transportation cost was estimated based on the cost per kilometer (km) covered, simulating the public transportation costs employed by the city halls. In this case, the estimated costs with depreciation, maintenance, vehicle insurances, driver wages, and toll fees were included. Therefore, the transportation cost was established based on the estimation of distances covered from the patients’ city of origin to the hospital where they are treated, considering a round trip.

### Home care cost

In order to estimate home care cost with the disease, a validated instrument was applied to the patient or his family via phone call. The questionnaire included questions about costs related to the acquisition of medications and medical materials, health insurance, required changes to the patient’s home, periodic consultations with experts outside the reference site, caretakers, and loss of patient’s productivity and of the relative’s productivity due to the disease (Supplemental material 1) [17].

### Cost analysis considering the clinical status of patients

The cost results were shown descriptively, considering the patient’s mobility status (wheelchair user or non-wheelchair user). The costs in the analyzed categories (hospital, home care, transportation, and total costs) for wheelchair users and non-wheelchair users were compared using the Mann-Whitney test, a non-parametric statistical test. Results were collected and are expressed in the Brazilian currency (Brazilian reais).

## Results

### Sample description

There were 43 follow-up patients identified in 2019 at the study site and, after evaluating for the inclusion criteria and invitation to participate, 27 patients were enrolled in the study (Supplemental Fig. 1). Table 1 shows the clinical characteristics of patients.

**Table 1:** Clinical characteristics of patients enrolled in the study

Clinical characteristics	Total patients (n = 27)
Age (years)	15.9 ± 5.5
Age at first symptoms (years)	3.4 ± 2.4
Age at diagnosis (years)	6.2 ± 2.7
Age at the start of any drug treatment (years)^a^	6.7 ± 3.6
Patients who are wheelchair users	17 (63%)
Age when started using wheelchair (years)	9.7 ± 2.0
Patients with scoliosis	15 (55%)
Patients using orthesis	8 (29%)
DMD stage^b^	
I - Pre-symptomatic	1 (4%)
II - Early ambulatory	7 (26%)
III - Late ambulatory (transitional phase)	2 (7%)
IV - Early non-ambulatory	14 (52%)
V - Late non-ambulatory	3 (11%)

^a^ Value calculated for the 23 patients taking medication to manage DMD. ^**b**^ Stage I: mild motor delay, mild muscle hypotonia, clumsy walking (from birth to 3 years of age); Stage II: presence of Gowers’ sign, toe walking, difficulty running, and difficulty climbing up or down stairs (2 to 7 years); Stage III: frequent falls, wheelchair use for long distances; Stage IV: wheelchair user, decreased function of arms, scoliosis, sits with no support from arms; Stage V: increased difficulty to use the upper limbs, restricted daily activities (from the end of adolescence to adulthood) [3, 8, 18].

Values indicated as n (%) or mean ± SD.

### Flow of hospital care

To evaluate the hospital costs, firstly the patient flow of care was mapped at the reference site. The follow-up of patients with DMD is conducted primarily in an outpatient setting and includes a series of periodic clinical appointments and exams (Supplemental Fig. 2).

### Estimating the costs per time unit and timepoints

The CCR were calculated for all the professionals categories and structural recourses (Supplemental Table 1) and used in the sequence to estimate the cost per resource per activity along the care pathway.

### Calculating the costs per patient

The total median cost (hospital, home care, and transportation) per patient/year was R$ 17,122 (IQR R$ 6,786; 25,621), within which 92% was of home care costs, 6% of hospital costs, and 2% of transportation costs. Table 2 shows the cost from all three categories of costs and its components. One patient was treated as an outlier for receiving drug treatment with ataluren and for showing a cost above three standard deviations of the sample. This patient showed an annual cost of R$ 994,758.

**Table 2:** Description of annual cost per category of cost^a^

Category of cost	Mean (SD)	Median (IQR25;75)	N
**Hospital (R$)**	1345 (1286)	1057 (515; 1899)	
Professionals	293 (232)	228 (89; 445)	26
Medications	1006 (1207)	767 (219; 1372)	21
Hospital structure	32 (35)	16 (5; 60)	26
Emergency care^b^	149 (35)	148	2
**Home care (R$)**	20,137 (23,452)	15,820 (5117; 25,096)	
Medications	1527 (1915)	1110 (0; 2370)	17
Food supplements	18 (94)	NA	1
Home infrastructure	6842 (19,701)	195 (0; 6700)	16
Transportation	335 (662)	0 (0; 404)	8
Health plan	912 (2239)	0 (0; 549)	7
Loss of productivity	9454 (11,391)	5932 (0; 15,106)	12
Ventilation	55 (282)	NA	1
Consultations outside the reference site	276 (733)	0 (0; 236)	9
Caretaker	715 (2910)	NA	2
**Transportation**	415 (426)	226 (103; 672)	
**Total (R$)**	21,898 (23,556)	17,122 (6786; 25,621)	

^a^ Consolidated results excluding the outlier patient. ^b^ The emergency care value includes costs with professionals and medications. ^c^ Patient and relative’s productivity.

SD: standard deviation; NA: not applicable; IQR25;75: first and third quartiles; N: number of patients who reported costs in the subcategories.

The composition of costs within the three classes (hospital, transportation, and home care) for every patient is shown in Supplemental Fig. 3. Among those patients who showed a proportionally higher hospital cost (P13, P20 and P25), two (P13 and P20) did not report home care costs, which contributes to the higher hospital proportion.

**Fig. 1:**
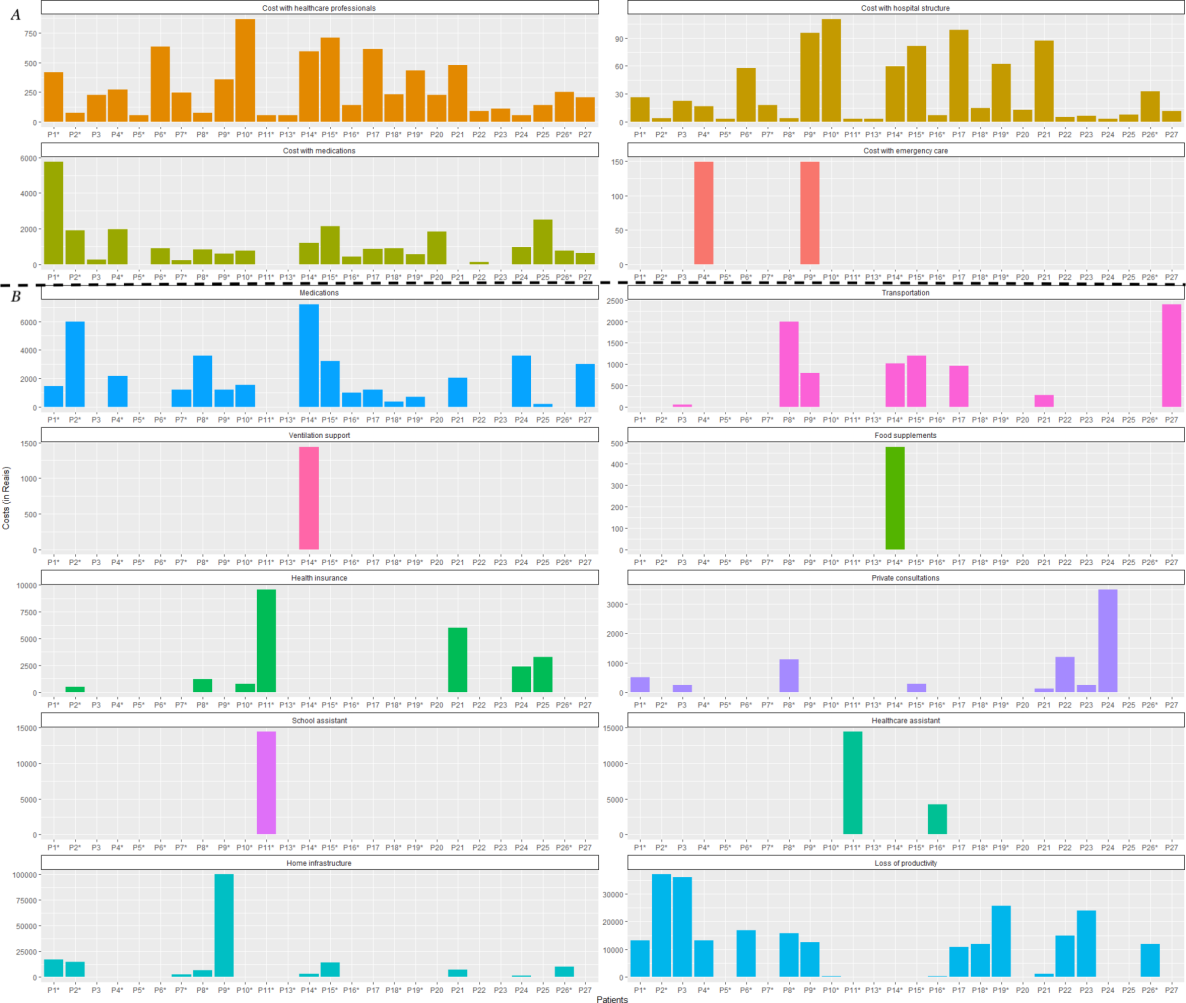
Composition of hospital and home care costs ^a^ ^a^**A** refers to the hospital cost and **B** refers to the home care cost for each patient. * Wheelchair user patient.

In order to understand the variability of sample costs, the composition of hospital and home care costs were evaluated separately. The cost items with the highest impact on the hospital costs were medications, with a median cost of R$ 767 (IQR R$ 219; 1,371). Among the categories analyzed in terms of home care costs, the median loss of productivity was the most impactful item, with median cost of R$ 5,932 (IQR R$ 0; 15,106), followed by medications, with median cost of R$1,110 (IQR R$ 0; 2,370). Figure 1A and 1B show the composition of hospital and home care costs, respectively. Three patients showed the highest hospital costs: P13, P20 and P25. Among these patients, P13 is a wheelchair user, while P20 and P25 are not wheelchair users, meaning it is not possible to directly associate a higher hospital cost to the fact a patient is a wheelchair user.

In the home care cost analysis, seven participating patients reported holding a health insurance plan (P2, P8, P10, P11, P21, P24, P25), with an annual mean cost of R$ 3,389 (SD R$ 3.331). In addition to the monthly fees of the plan, this group of patients also reported an annual mean cost of R$ 677 (SD R$ 1,312) with private medical and non-medical consultations. Among patients with no health insurance plan, the annual mean cost with private consultations was R$ 105 (SD R$ 282).

Overall, 23 categories of professionals were mentioned among the consultations conducted in 2019 outside the reference site. The professional category with the highest number of consultations attended by patients was motor physical therapist (with 1,840 consultations reported by 22 patients) (Table 3).

**Table 3:** Consultations performed in 2019 outside the reference site

Professional category^a^	Consultation (n)	Patients (n)
Motor physical therapist	1840	22
Speech therapist	624	10
Psychologist	445	12
Respiratory physical therapist	432	7

^a^ Other professional categories mentioned: physiatrist, aquatic physical therapist, nutritionist, gastroenterologist, cardiologist, geneticist, neurologist, pulmonologist, endocrinologist, occupational therapist, orthopedist, urologist, psychiatrist, traumatologist, social worker, ophthalmologist, pedagogue, pediatrist, swimming instructor.

### Analyses between groups

**Fig. 2:**
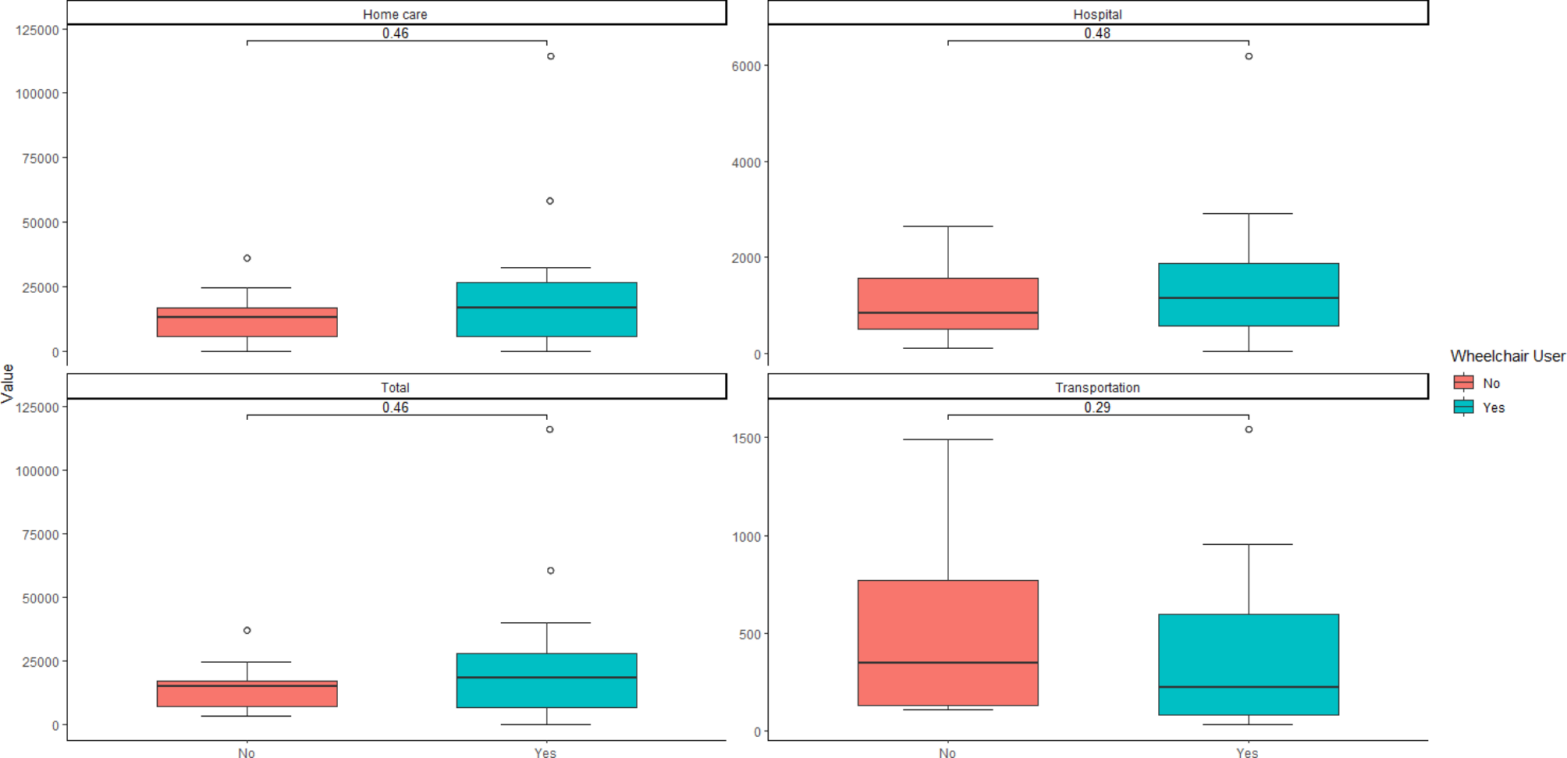
Comparison of home care, hospital, transportation, and total median costs between wheelchair users and non-wheelchair users

No significant difference was observed between the median costs for wheelchair users and non-wheelchair users. Descriptively, the median cost for wheelchair users was 22.63% higher than that for non-wheelchair users, with the highest difference being explained by the hospital costs (Fig. 2).

## Discussion

The economic burden associated to the care provided to subjects with rare diseases is significant worldwide, and there is still a need for studies that measure the use of resources by these patients in the health system. In addition, researches estimating the economic impact under the perspective of this system vary in terms of methodology, which makes it difficult to use this information to manage resources [19]. This is a pioneer study in Latin America for measuring cost using the micro-costing technique for patients with DMD. The use of human, structural and health technologies resources was measured for patients with the disease over a period of one year, and it was identified that home care costs are more significant than hospital costs, and that wheelchair users have a higher cost to deal with. The information generated may help supporting health policies in rare diseases based on scientific evidence from economic data.

The observed median annual cost of R$ 17,122 (representing a PPP, Purchasing Power Parity of 7,285) is close to the data estimated in a North American study (8,735 dollars a year) that evaluated the expected cost in 20 years for patients with the disease diagnosed at five years of age and with loss of the ability to walk by the age of 11 treated with prednisone [20]. In the payers point of view and using the micro-costing technique, in Germany, the direct yearly costs for patients with DMD were estimated to be 28,951 Euros (PPP 39,604), with 46% of this cost being justified by hospitalizations, rehabilitation services, and investments in housing [17]. Despite the differences in values, which can be partially explained by the different methodologies adopted and pricing policies, the European study also showed an increase in costs as the severity of the patient’s disease increased, with the costs for patients who are no-ambulatory (stage V) being six times higher than those for children in ambulatory transitional phase (stage III). In both studies, the analyses were performed based on payment data, without evaluating the use of resources per patient as in the micro-costing studies, in addition to including distinct variables.

The care for DMD is mostly provided by multidisciplinary teams and, therefore, is concentrated in specialized care sites. In Brazil, patients with DMD have access to treatment by the public health system, SUS, and their families are entitled to the benefits foreseen in law for subjects with disability. The use of the information generated, which shows the financial impact for the patients’ families, indicate the importance of establishing economic strategies for rare diseases involving the State, treatment sites, and organizations that provide therapeutic technologies, so that they can be properly used, resulting in better health outcomes.

The opportunity to understand the variability in the use of health system resources due to the health status of every patient provided by micro-costing methods helps establishing health policies focused on the actual needs of patients. Measuring the incremental costs related to the loss of the ability to walk has a significant contribution to the evaluation process for health technologies in DMD. Technologies that have been recently approved by the Food and Drug Administration (FDA), eteplirsen (Exondys) and golodirsen (Vyondys 53) have the benefit of delaying the loss of the ability to walk [20, 21]. Based on the study conducted using the TDABC method, the national evaluations regarding the incorporation of these technologies in the Brazilian health system may use information that show the actual cost associated with the anticipated worsening of the health status of patients who have no access to this technology.

Some precautions must be considered when using the cost information generated in this study. The inclusion of patients from a single center is the main one. Thus, generalizations about the information presented both nationally and internationally must be made with caution. However, it must be observed that the molecular epidemiology of the patients in this study is similar to that of the largest Brazilian casuistry described recently (Supplemental Table 2) [6].

The presence of a single patient on ataluren treatment in the sample made it impossible to conduct evaluations showing the incremental cost related to the use of new drugs that restore the expression of dystrophin in specific patient profiles. Finally, this study focused on the description of costs from a patient cohort, and future analyses might use the data presented herein to build economic models to evaluate health technologies incorporation and remuneration for DMD in the health system.

## Conclusion

The care of patients with DMD represents a significant cost that is primarily justified by family disbursement (92%), followed by hospital costs. This is the first study conducted in Latin America measuring the treatment costs for follow-up patients with DMD in the Brazilian health system, contributing to the international literature, which is scarce in reporting data on the use of resources in the health system by patients with rare diseases. Generating accurate information about costs is crucial to provide health managers with information that make it possible to establish more sustainable policies when managing rare diseases in emerging countries.

## Electronic supplementary material

Below is the link to the electronic supplementary material.

Supplementary Material 1: This document includes the “Instrument for collecting household expenses and indirect costs”, and “Equations of loss of patient’s productivity”.

Supplementary Material 2: Supplementary Fig. 1: Flowchart of patient inclusion.

Supplementary Material 3: Supplementary Fig. 2: Macro-flow of care for patients with Duchenne Muscular Dystrophy at a specialized site.

Supplementary Material 4: Supplementary Fig. 3: Composition of costs for each patient enrolled according to the categories of cost.

Supplementary Material 5: Supplementary Table 1: Cost-capacity-rate (CCR) for professionals.

Supplementary Material 6: Supplementary Table 2: Frequency of mutation among the study patients compared to Brazilian population reported by Almeida et al., 2017 [6].

## Data Availability

The datasets used and/or analyzed during the current study are available from the corresponding author on reasonable request.
