# Supplementary material for: Estimated costs for Duchenne muscular dystrophy care in Brazil
Source: Orphanet J Rare Dis. 2023 Jun 22;18:159. doi: 10.1186/s13023-023-02767-6 (PMC10288739; doi:10.1186/s13023-023-02767-6)
Supplement: Supplementary file 1 — Supplementary Material 1: This document includes the “Instrument for collecting household expenses and indirect costs”, and “Equations of loss of patient’s productivity”. [file 13023_2023_2767_MOESM1_ESM.docx]

# Supplementary material

#

# Instrument for collecting household expenses and indirect costs:

This questionnaire can be answered by the person with the disease or responsible for the patient with the disease.

The management of patients with Duchenne muscular dystrophy is carried out in SUS referral centers. However, we know that some additional expenses may exist that are not covered by the SUS. In this sense, the questions below aim to estimate the monthly expenditure related to the disease by the patient's family (direct family group) (questions 1 to 5) and the impact related to lost productivity (questions 6 to 15).

Under no circumstances will responses be shared with any identification.

# - Due to Duchenne muscular dystrophy, what is the patient's monthly expenditure on medication before the pandemic?

1. **– Before the pandemic, did the patient have health insurance?**

*If the answer is yes:*

What is the monthly cost of the health insurance?

# A- Did the patient have a routine of monthly consultations with which professionals before the pandemic? Note: Indicate all professionals in which consultations are held monthly. The professionals in which consultations are held annually will only be indicated in the next question.

physiatrist

motor physical therapist respiratory physiotherapist aquatic physical therapist Nutritionist

Psychologist speech therapist Gastroenterologist Cardiologist geneticist Neurologist pulmonologist Endocrinologist

Occupational therapy

Other. Which?

*For each professional indicated in the previous question, ask:*

# Number of monthly consultations:

**Monthly cost (R$):**

# B - Did the patient have a routine of annual consultations with which professionals before the pandemic? Note: Indicate all professionals in which consultations are held annually.

physiatrist

motor physical therapist respiratory physiotherapist aquatic physical therapist Nutritionist

Psychologist speech therapist Gastroenterologist Cardiologist geneticist Neurologist pulmonologist Endocrinologist

Occupational therapy

Other. Which?

*For each professional indicated in the previous question, ask:*

# Number of monthly consultations:

**Monthly cost (R$):**

# A – Before the pandemic, did you and/or your family need a caregiver to assist in your daily life at home?

*If the answer is yes:*

Monthly cost (R$)?

# B - Before the pandemic, did you and/or your family need a caregiver to assist in your day- to-day with school activities?

*If the answer is yes:*

Monthly cost (R$)?

# – Before the pandemic, did the family need to make any investment in housing infrastructure to meet the needs of the Duchenne muscular dystrophy patient? (examples: special bed, wheelchair, equipment to assist in feeding)

*If the answer is yes:*

What and how much was invested (R$)?

Patient productivity loss:

1. **A - Does the patient perform, or has he performed, a professional activity?** *If the answer is yes:*

# 6 B - Before the pandemic (2019), did the patient miss work due to Duchenne muscular dystrophy? *If the answer is yes:*

1. **C - How many days did the patient stop working (missed work) due to Duchenne muscular dystrophy before the pandemic (2019)?**

# - What is the patient's monthly reference salary? (Consider the last salary base of 2019)

*(Ask if the answer 6a was yes)*

# - Did the patient have any salary reduction because of absences resulting from the disease before the pandemic (2019)?

*If the answer is yes:*

What is the percentage of reduction?

1. **- How many months was there a salary reduction in 2019?** *(Ask if the answer 8 was yes)*

# 10- Did the patient have to stop working permanently?

*If the answer is yes:*

With which age?

Family productivity loss:

# 11 A - Does the family member exercise (or has he/she already exercised) a professional activity? *If the answer is yes:*

**11 B - Before the pandemic (2019), did the family member miss work due to Duchenne muscular dystrophy?** *If the answer is yes:*

# C- How many days did the family member stop working (missed work) due to Duchenne muscular dystrophy before the pandemic (2019)?

1. **– What is the family member's monthly reference salary? (Consider the last salary base of 2019)** *(Ask if the answer 11a was yes)*
2. **- Did the family member have any salary reductions due to absences resulting from the disease before the pandemic (2019)?** *(Ask if the answer 11b was yes)*

*If the answer is yes:*

# What is the percentage of reduction?

1. **- How many months was there a salary reduction in 2019?** *(Ask if the answer 13 was yes)*

# 15- Did the family member have to stop working permanently?

*If the answer is yes:*

With which age?

# Equations of loss of patient’s productivity:

**Loss of patient’s productivity = PP(inability) + PP(disability)** (Equation 1) Where:

PP = Loss of productivity and

**PP(inability) = (AbsLP + AbsCP + Rehab) x Sa + Slost x t**

AbsLP = Long-term absence due to DMD in one year (days)

AbsCP = Short-term absence (hours) due to DMD in one year (hours converted into days) Rehab = Rehabilitation days throughout one year

Sa = Current salary per day

Slost = Salary loss per month caused by changes to decreased workload T = Number of months with decreased salary due to changes

**PP(disability) = Dlost x Sdi**

Dlost = Days of work lost due to the disease

Sdi = Last salary reference per day adjusted by the current inflation (if the patient has never worked, a national minimum wage reference must be considered)

**Loss of relative’s productivity = LP(absence) + LP(changes)** (Equation 2) Where:

LP = Long term And:

**LP(absence) = (AbsLP + AbsCP + Rehab) x Sd**

AbsLP = Long-term absence due to DMD in one year (days)

AbsCP = Short-term absence (hours) due to DMD in one year (hours converted into days) Rehab = Rehabilitation days throughout one year

Sd = Current salary per day

**LP(changes) = Slost x t + Dlost x Sdi**

Slost = Salary loss per month caused by changes to decreased workload t = Number of months with decreased salary due to changes

Dlost = Days of work lost due to the disease

Sdi = Last salary reference per day adjusted by the current inflation (if the patient has never worked, a national minimum wage reference must be considered)
