# Supplementary figures and images for: Estimated costs for Duchenne muscular dystrophy care in Brazil
Source: Orphanet J Rare Dis. 2023 Jun 22;18:159. doi: 10.1186/s13023-023-02767-6 (PMC10288739; doi:10.1186/s13023-023-02767-6)

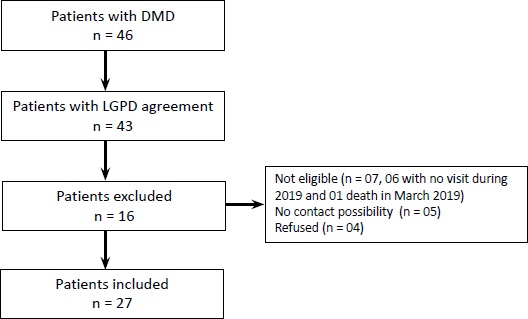

Supplement: Supplementary file 2 — Supplementary Material 2: Supplementary Fig. 1: Flowchart of patient inclusion. [file 13023_2023_2767_MOESM2_ESM.jpg]

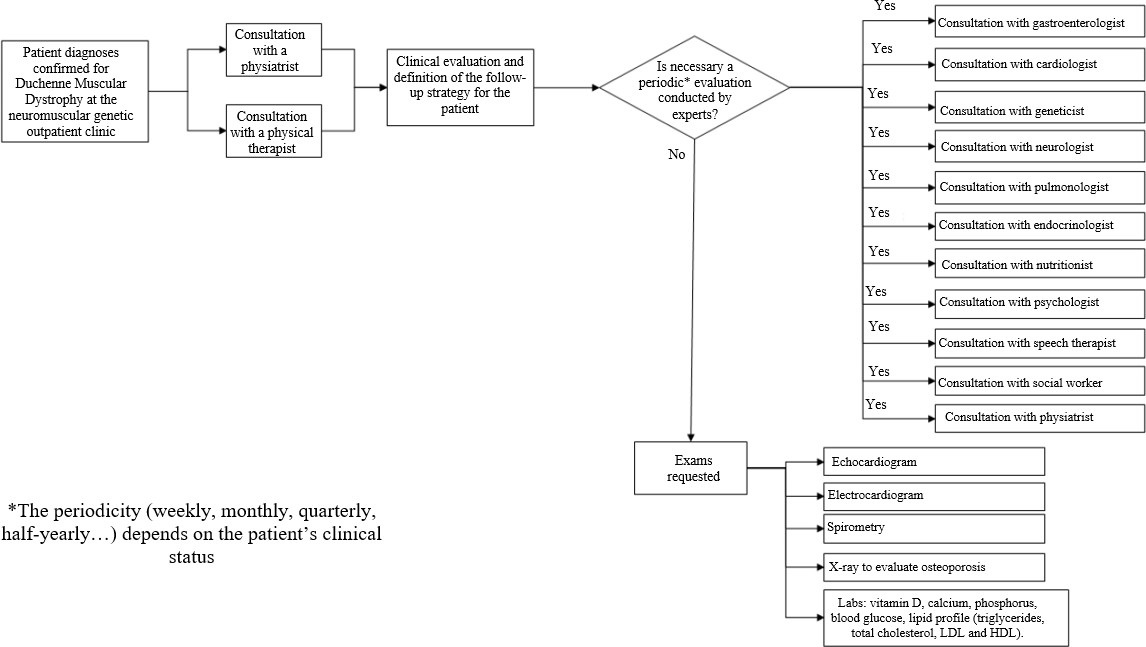

Supplement: Supplementary file 3 — Supplementary Material 3: Supplementary Fig. 2: Macro-flow of care for patients with Duchenne Muscular Dystrophy at a specialized site. [file 13023_2023_2767_MOESM3_ESM.jpg]

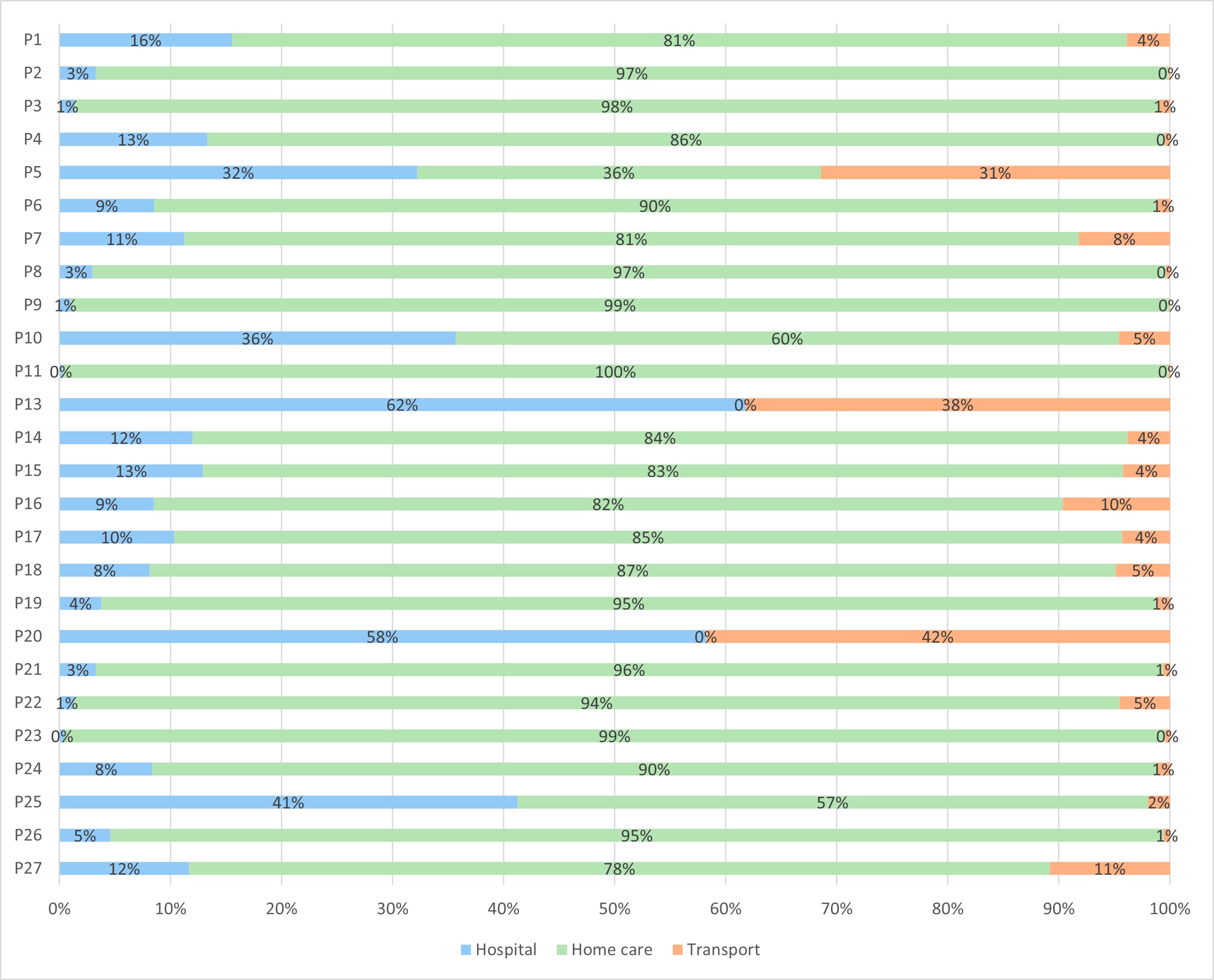

Supplement: Supplementary file 4 — Supplementary Material 4: Supplementary Fig. 3: Composition of costs for each patient enrolled according to the categories of cost. [file 13023_2023_2767_MOESM4_ESM.jpg]
