# Supplementary material for: Estimated costs for Duchenne muscular dystrophy care in Brazil
Source: Orphanet J Rare Dis. 2023 Jun 22;18:159. doi: 10.1186/s13023-023-02767-6 (PMC10288739; doi:10.1186/s13023-023-02767-6)
Supplement: Supplementary file 5 — Supplementary Material 5: Supplementary Table 1: Cost-capacity-rate (CCR) for professionals. [file 13023_2023_2767_MOESM5_ESM.docx]

**Table 1** Cost-capacity-rate (CCR) for professionals.

| **Professional class** | **Mean time (hours)** | **CCR (R$/hour)** |
| --- | --- | --- |
| **Professional resources**  Neurologist | 0.5 | 113 |
| Physiatrist | 0.67 | 113 |
| Cardiologist | 0.33 | 127 |
| Physical Therapist | 0.67 | 59 |
| Gastroenterologist | 0.25 | 113 |
| Geneticist | 0.5 | 111 |
| Pulmonologist | 0.33 | 113 |
| Endocrinologist | 0.33 | 113 |
| Nutritionist | 0.66 | 58 |
| Psychologist | 0.75 | 56 |
| Speech Therapist | 0.66 | 58 |
| Occupational Therapist | 0.66 | 59 |
| Nurse | 0.5 | 58 |
| **Outpatient structure resources** |  |  |
| Outpatient care rooms | NA | 6 |

NA: Not applicable.
