# Supplementary material for: Estimated costs for Duchenne muscular dystrophy care in Brazil
Source: Orphanet J Rare Dis. 2023 Jun 22;18:159. doi: 10.1186/s13023-023-02767-6 (PMC10288739; doi:10.1186/s13023-023-02767-6)
Supplement: Supplementary file 6 — Supplementary Material 6: Supplementary Table 2: Frequency of mutation among the study patients compared to Brazilian population reported by Almeida et al., 2017 [6]. [file 13023_2023_2767_MOESM6_ESM.docx]

**Table 2** Frequency of mutation among the study patients compared to Brazilian population reported by Almeida et al., 2017 (6).

| **Type of mutation** | **Total of patients**  **(n = 27)** | **Almeida et al., 2017**  **(n = 148)** |
| --- | --- | --- |
| **Frameshift** | 1 (3,7%) | 10,8% |
| **Large duplications** | 2 (7,4%) | 10,1% |
| **Large deletions** | 19 (70,4%) | 60,8% |
| **Nonsense** | 2 (7,4%) | 12,8% |
| **Splicing** | 3 (11,1%) | 4,0% |

Values indicated as n (%).
